# A movable beast: glaciation in the Ediacaran

**DOI:** 10.1093/nsr/nwad153

**Published:** 2023-05-25

**Authors:** Joseph L Kirschvink

**Affiliations:** Division of Geological & Planetary Sciences, California Institute of Technology, USA; The Marine Core Research Institute, Kochi University, Japan

Ediacaran time (635–543 million years ago [Ma]) represents the dawn of macroscopic life following two Neoproterozoic Snowball Earth episodes [1], where a runaway ice-albedo effect caused the entire planet to become engulfed in ice [2,3]. (During Phanerozoic time, glaciation on Earth was limited to mid-to-high latitudes [4].) Large fossils resembling animals first appeared shortly after the last Snowball [5] but questions remain about the environment in which animal life first took hold. An enduring Ediacaran enigma is the occurrence of multiple glacial deposits on multiple continents. Were they synchronous? Were they brief Snowballs? Did the retreat of glaciation spark animal life [1]?

To answer these questions, we must know the style of glaciation. In a paper published in this Issue, Wang *et al.* [6] analysed the carbon isotopes of the strata directly underlying the Tarim glacial deposits and discovered a diagnostic signal that is useful for global correlation: the large-amplitude and long-lived ‘Shuram’ isotope excursion. This excursion has been dated at 570–560 Ma [7], thus establishing an age of <560 Ma for the Tarim glaciation. But the age could not have been more different from the only other well-dated Ediacaran glacial deposits—the ∼580 Ma Gaskiers glaciation from Canada [8]. Considering these two ages of glaciation alone, Wang *et al.* could see that Ediacaran glaciation had occurred at least twice, not once, separated by as much as 20 million years.

They then devised a clever way of predicting ages on the ∼33 other glacial deposits using two simple rules. The first was to assume that there were no Ediacaran ‘Snowball’ glaciations, so the deposits would only have formed at mid-to-high latitudes. The second rule was that the continents had rotated largely *en masse* due to true polar wander (TPW) [9,10]. TPW is when a planet ‘tips over’ to keep most of its mass anomalies on the equator, much like your arms are drawn out when you spin yourself. There is ample evidence for TPW during Ediacaran and early Cambrian times [9,11,12], as all major continental fragments show large, great-circle arcs in the location of their ancient rotational axes determined through paleomagnetic studies. In fact, by moving the bits of Gondwana around on a sphere until these arcs line up, one can recreate the well-known Ediacaran–Cambrian assembly of Gondwana [9]. Alfred Wegner would have been pleased!

**Figure 1.**
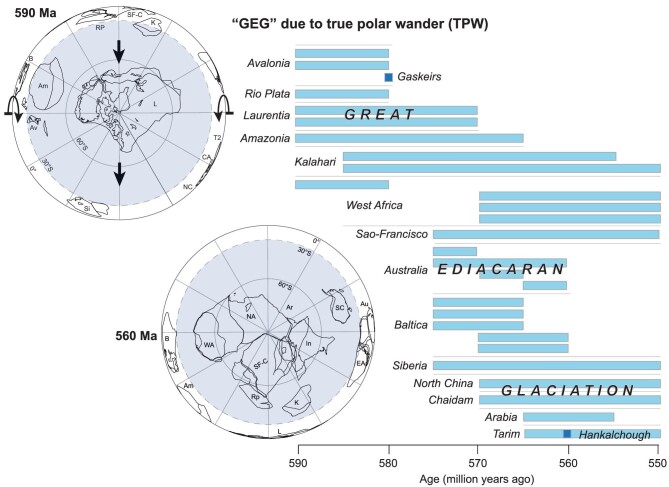
**Figure 1.** Continuous but diachronous Ediacaran glaciation during a ∼90˚ rotation of true polar wander (TPW) as different continents moved through different latitudinal climate zones at different times. The 590- and 560-Ma globes show the before and after continental positions [10]. View from the South Pole, where some continents cannot be seen in the northern hemisphere. Figure courtesy of R. Mitchell.

Wang *et al.*’s [6] resulting Ediacaran glacial model is striking (Fig. 1). Continents would become glaciated as they rotated through mid-to-high latitudes and their ice sheets would melt when they moved into the tropics. There was just one, continuous ice age—the ‘Great Ediacaran Glaciation’—but it was a moving target that occurred on different continents at different ages. Ages can now be predicted based on their paleogeography!

Numerous other tests of this novel hypothesis are also possible, such as the fact that sea-level rises should happen at localities that move towards the equator but those approaching the poles should experience sea-level drops [13]. However, perhaps the authors have not gone far enough: I would step back and ask whether there was a central, unifying process that might be the driving mechanism for all these weird events during Ediacaran time. For example, the second-largest superplume in Earth History, the Sept-Îles ultramafic intrusive complex in the Gulf of St. Lawrence, Canada, erupted right in the middle of this mess. In addition to helping excite TPW, it could explain deeply excised marine canyons in Australia and Laurentia, and perhaps perturb the marine ecosystem enough to generate the Shuram Excursion itself. The dartboard of these hypotheses is large enough to keep a good fraction of the Earth Science community shooting at them for a long time.
